# Postoperative Choroidal Vascular Biomarkers in Eyes with Rhegmatogenous Retinal Detachment-Related Giant Retinal Tears

**DOI:** 10.1186/s40942-023-00482-9

**Published:** 2023-08-01

**Authors:** Miguel A. Quiroz-Reyes, Erick A. Quiroz-Gonzalez, Miguel A. Quiroz-Gonzalez, Virgilio Lima-Gomez

**Affiliations:** 1 Retina Department of Oftalmologia Integral ABC, Medical and Surgical Assistance Institution (Nonprofit Organization) Affiliated with the Postgraduate Studies Division at the National Autonomous University of Mexico, Av. Paseo de las Palmas 735 Suite 303, Lomas de Chapultepec, 11000 Mexico City, Mexico; 2 Institute of Ophthalmology. Fundacion Conde de Valenciana, Medical and Surgical Assistance Institution (Nonprofit Organization) Affiliated with the Postgraduate Studies Division at the National Autonomous University of Mexico, Calle Chimalpopoca 14. Col Obrera, 06800 Mexico, Mexico; 3 Juarez Hospital, Public Assistance Institution (Nonprofit Organization), Av. Politecnico Nacional 5160, Colonia Magdalena de las Salinas, 07760 Mexico City, Mexico

**Keywords:** Choriocapillaris flow area, Choroidal vascularity index, Giant retinal tear, Rhegmatogenous retinal detachment

## Abstract

**Purpose:**

Choroidal vascularity index (CVI) and choriocapillaris flow area (CFA) are perfusion biomarkers relevant to retinal disease management. There is limited knowledge regarding these biomarkers in eyes that have been successfully treated for rhegmatogenous retinal detachment (RRD) due to giant retinal tears (GRTs). This study aimed to analyze the relationship between choroidal perfusion biomarkers and functional outcomes in surgically treated eyes with GRT-associated RRD and their fellow eyes.

**Methods:**

A total of 33 GRT eyes and 29 fellow eyes were included in this study. All RRD-GRT eyes were treated with vitrectomy and categorized into two groups based on whether additional scleral buckles (SB) were placed. Visual and choroidal features were compared between the groups.

**Results:**

The subjects had an average age of 55.18 years, a mean time of 2.36 weeks before surgery, and a mean follow-up time of 25.9 months. Best-corrected visual acuity (BCVA) was substantially worse in GRT eyes (1.9 logMAR) than in fellow control eyes (0.23 logMAR) but substantially improved after surgery (0.59 logMAR). There were no differences in the presurgical characteristics and BCVA between the eyes that did and did not undergo SB. Long-term CVI and CFA were lower in eyes with GRT than in their fellow eyes. Among eyes with GRT, those with SB had significantly lower CVI and CFA. Correlation analysis revealed that the CVI and CFA were positively correlated with visual outcomes (negative correlation with logMAR).

**Conclusion:**

Despite successful surgical repair, long-term functional and choroidal evaluations showed permanent changes in eyes with GRT. Positive correlations between perfusion biomarkers and visual function suggest that better choroidal vasculature is associated with better visual outcomes. The results of this study highlight the benefits of analyzing choroidal vasculature biomarkers and the relationship between the choroidal anatomy and vision.

**Supplementary Information:**

The online version contains supplementary material available at 10.1186/s40942-023-00482-9.

## Key message

1. Surgical intervention for GRT-associated RRD can significantly improve vision; however, its management remains challenging.
2. Buckled eyes with GRT-associated RRD showed substantial alterations in choroidal vasculature relative to non-buckled eyes and fellow control eyes.

**Supplementary Information:**

The online version contains supplementary material available at 10.1186/s40942-023-00482-9.

## Background

Rhegmatogenous retinal detachment-related giant retinal tears (RRD-related GRTs) are characterized by a full-thickness circumferential retinal break of at least 90° [1] that may cover more than 180° in some cases [2], accompanied by a detached vitreous in the posterior region [1, 2]. The occurrence of this condition is uncommon, but it is associated with significant visual loss and proliferative vitreoretinopathy (PVR) [3–5]. GRT-associated RRD is typically initiated by liquefaction of the central vitreous gel, which leads to shrinkage of the surrounding vitreous and a pull force at the vitreous base. The tractional force subsequently evolves into a tear at the posterior vitreous base and a neurosensory break stretching around the retina [1, 3]. Compared to the posterior region, the vitreous remains attached to the anterior flap [1]. Therapeutic interventions for GRT-associated RRD include cryotherapy, laser photocoagulation, and surgery to reattach the neurosensory connection of the detached retina to the retinal pigment epithelium (RPE) by reducing tension along the vitreous base [4].

There are several surgical options for GRT, including three-port pars plana vitrectomy (PPV) and scleral buckling (SB) placement [3, 6, 7]. Although these procedures are frequently successful in correcting GRT, complications can develop, making them difficult to manage [3]. PVR, which affects 40–50% of all GRT cases, is a common cause of postsurgical complications and is particularly prevalent in trauma-associated GRT [8]. PVR can lead to poor visual outcomes, despite the high rate of anatomical success after reattachment [3, 9, 10].

PPV combined with SB (PPV + SB) is regarded as one of the preferred surgical options to repair retinal breaks and relieve vitreoretinal traction in GRT-associated RRD [11]. This technique is used to treat several different representations of retinal detachment (RD) with an impressive single-operation success rate (SOSR) [12]. PPV + SB is particularly preferred for GRT-associated RRD cases characterized by an excessive number of breaks or long breaks or in cases where vitrectomy techniques alone cannot completely release vitreous traction [13]. In recent years, PPV without SB has gained popularity, with several studies suggesting that this technique is equal to or more effective than PPV + SB [14, 15]. The use of PPV without SB has been reported to have high anatomical success rates in RD repair in general [16], but others have argued that a combination of both techniques (PPV + SB) could provide superior surgical outcomes and achieve a higher SOSR [17].

Beyond anatomical outcomes, surgical success can be evaluated based on visual outcomes, mainly the best-corrected visual acuity (BCVA). Since the introduction of modern imaging techniques such as optical coherence tomography angiography (OCT-A), it has become popular to collect perfusional changes and correlate them with anatomical and visual outcomes [18–20]. Multiple studies applied OCT-A to investigate alterations in retinal structure and perfusion to establish correlations with visual outcomes [19, 21, 22]. Choroidal thickness and choriocapillaris flow area (CFA) are biomarkers that have been investigated as indicators of retinal health [23–29].

Recently, the choroidal vascularity index (CVI) has been introduced as a new vascular biomarker to assess perfusional and vascular flow in the choroid. CVI is the percentage of vascular luminal area (LA) within the preselected total choroidal area (TCA) [30]. A recent study reported a decrease in retinal and choroidal perfusion after PPV with or without SB, which was more significant in eyes with SB [31]. In addition, SB has been associated with a decrease in retinal arterial blood flow rate [32], but its effects on CVI and CFA have not been previously established [33]. Furthermore, reports on the correlation between choroidal biomarkers and visual function in GRT-associated RRD treated with PPV and SB are limited.

This study quantitatively evaluated postoperative CVI and CFA after a long follow-up period in patients who underwent vitrectomy alone or in combination with scleral buckling. The findings of this study provide insights into the effects of vitrectomy and SB on CVI and CFA. The relationships among CVI, CFA, visual function, and other postoperative outcomes for GRT-associated RRD were also explored.

## Methods

### Study design

This study followed the principles of the Declaration of Helsinki and was approved by our institutional ethics committee. All participants provided written informed consent to participate in this study.

In this study, patients’ medical records, which were retrospectively studied to compare the retinal and choroidal perfusion, vessel densities, and structural outcomes of interventions in RRD-related GRT cases, were reviewed to collect postoperative CVI, CFA, and CSFT values in the surgical group. This was the same group of patients previously published [31]. The same values for the contralateral (fellow) eyes were used as the control group. The primary objective of this study was to quantitatively evaluate and statistically compare macular CVI, CFA, and CSFT scores, and their correlations with visual changes. The secondary outcomes included investigating the effect of SB on choroidal perfusion markers by comparing the values in both subsets of surgically treated eyes to those obtained from the control group.

The inclusion criteria were non-randomly selected RRD-related GRT cases successfully treated with PPV alone or PPV in combination with SB. Patients were included if they were at least 18 years old and had a best-corrected visual acuity (BCVA) at the last visit of 1.60 logMAR or lower, complete anatomical and structural retinal reattachment only in the selected eyes, and no signs of silicone oil at the final postoperative visit (minimum 6-month follow-up period). Additional criteria included the availability of perfusion, and functional and structural assessments at the last clinical examination.

Patients with the following characteristics were excluded: a previous record of surgical complications, GRT-associated RRD exhibiting macular hole RD, PVR associated with recurring RRD, and the presence of active glaucoma. In addition, participants who were lost to follow-up were also excluded. Similarly, patients with critical complications (e.g., endophthalmitis) were excluded. Overall, 33 eyes that met the inclusion criteria were selected and classified based on the surgical procedure used to correct GRTs.

### Examinations

A detailed preoperative evaluation and detailed methods performed have been described previously [34], including the measurement of visual acuity, slit-lamp assessment, fundoscopy, and indirect ophthalmoscopy. Spectralis OCT (Heidelberg Engineering, Heidelberg, Germany) was used to capture horizontal images through the foveal center. Postoperative perfusion and choroidal flow evaluations were performed using an OCT angiography device (RTVue XR Spectral Domain OCT Avanti with AngioVue Software, OptoVue Inc., Fremont, CA, USA). Partial-coherence laser interferometry was performed using a Zeiss IOL Master 700 (Carl Zeiss Meditec AG, Oberkochen, Germany) to measure axial length. Pathological conditions were diagnosed using a combination of B-scan ultrasonography (A and B Ultrasound Units; Quantel Medical, Du Bois Loli, Auvergne, France) and indirect ophthalmoscopy. Postoperative structural, functional, and perfusion evaluations were performed during the follow-up assessments. In all the patients, postoperative CVI and CFA of the macula were performed during the final evaluation.

### Surgical protocol

The surgical techniques used to treat these patients have been previously described [31, 34]. Conventional 25-gauge three-port pars plana vitrectomy (PPV) techniques were used. During the procedure, a diluted suspension of triamcinolone acetonide (Kenalog 40 mg/mL; Bristol-Myers Squibb, New York, NY, USA) was used to improve visualization of the posterior hyaloidal condensation (PHC) and its base. Active suction was used to pull the PHC from the superficial retina via a soft-tipped microcannula before the use of perfluorocarbon heavy liquid injection to hydropneumatically reattach the retina and drain the subretinal fluid (SRF), followed by argon laser endophotocoagulation after complete retinal reattachment. A low-lying SB was placed in a subset of cases to support the retinal edge and release the residual traction. These cases were selected based on the following findings: inferior location of the GRT, evidence of PVR grade C or worse, GRT cases with a circumferential tear extension of less than 180º, and the presence of other risk factors such as trauma, young age, high myopia, and hereditary conditions such as Marfan syndrome and Stickler syndrome [35, 36]. Finally, a 15% octafluoropropane (C_3_F_8_) gas mixture or 5000 cs silicone oil were added as a long-lasting tamponade.

### Image binarization for CVI quantification and CFA measurement

**Fig. 1:**
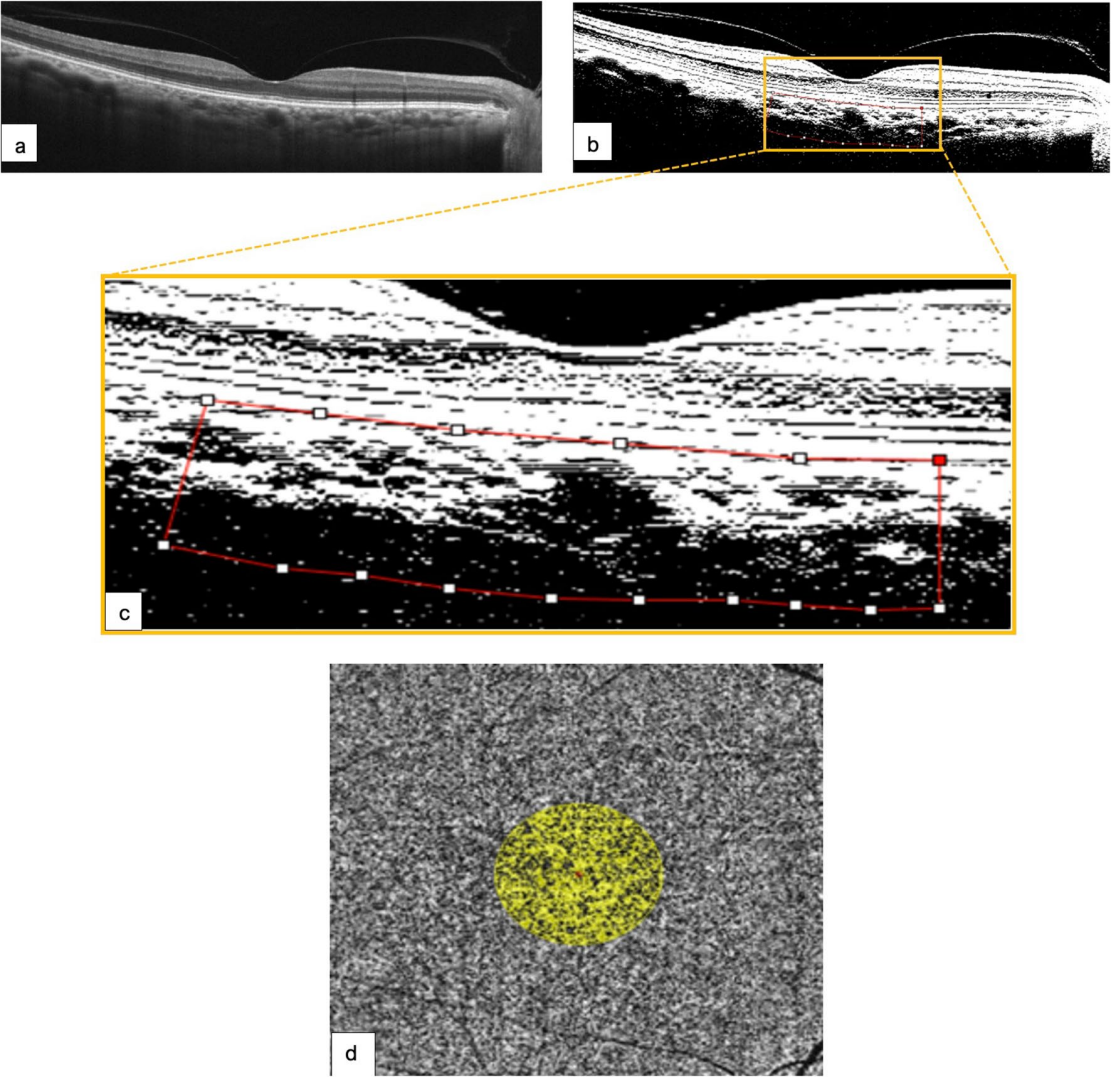
Control of normal eyes. **a** High-definition 9-mm horizontal B-scan designed to depict the intraretinal structure and subfoveal choroidal layers in a normal eye in greater detail. **b** Enhanced B-scan image with binarized processing of the subfoveal choroidal stroma and luminal vascular visualization of the subfoveal choroidal vessels to obtain the choroidal vascularity index (CVI). The red dotted line clearly delineated the selected subfoveal area. **c** Magnified image from the yellow insert depicts a choroidal vascularity index (CVI) of 69.8%, calculated within the area clearly delineated by the red dotted line. **d** A normal choriocapillaris flow area (CFA) of 2.348 mm^2^ in the selected evaluation area of 3.142 mm^2^

To calculate the CVI values, the luminal area (LA) and total choroidal area (TCA) were quantified from the enhanced SD-OCT images of the macula using the ImageJ analysis software (version 1.53; NIH, Rasband and contributors, USA, public domain). OCT-B images (9-mm horizontal) were uploaded and converted to an 8-bit format (Fig. 1a) and then adjusted using the autothreshold technique (Niblack autothreshold). Then, using the polygon tool, the area of the subfoveal choroid was manually selected to map the total choroidal area (TCA) from 750 µm nasal to temporal in the direction of the horizontal plane from the foveal center and vertically from the RPE-Bruch’s membrane region to the inner scleral border in the direction of the vertical plane (demarcated by the dotted red line in Fig. 1b). Subsequently, the stromal vascular tissue area was determined by the number of white pixels, and the LA at the enhanced choroid was determined by applying the threshold tool to quantify the number of dark pixels once the binarized image was converted back to the RGB space (Fig. 1c). Finally, the dark-to-light pixel ratio was expressed as a percentage and defined as CVI (= (LA/TCA) × 100), as previously described by Agrawal et al. [30, 37]. The protocol study method for binarization is shown in Fig. 1 and has been validated in a previous publication [38]. The CFA was obtained by automated binarization and segmentation of the choriocapillaris subfoveal plexus slabs using the RTVue XR OCT Avanti with AngioVue Software (OptoVue Inc., Fremont, CA, USA) and automatically calculated from a 3.142 mm^2^ evaluation area (Fig. 1d).

### Statistical analysis

Data were collected, checked for accuracy, and transferred to GraphPad Prism (version 9.2.0) and R software (version 4.1.1) for statistical analysis. Using the Shapiro–Wilk test, data were checked for normal distribution and appropriate tests were selected. The Wilcoxon paired sign rank test was used to identify differences in axial length, preoperative BCVA, postoperative BCVA, and choroidal parameters between surgical and contralateral eyes. The Chi-square test was used to test for differences between the surgical groups in terms of additional surgeries performed and the tamponade used. The two-tailed Mann–Whitney U test was used to identify differences in BCVA and choroidal parameters between the surgical cohorts in terms of presurgical characteristics. Correlations between BCVA and choroidal perfusion markers were tested using Pearson’s correlation coefficient. The significance cutoff for the tests was set at p < 0.05. A multivariate linear regression model was created in the R environment using the *LM* function.

## Results

### Characteristics of the eyes

A total of 33 eyes with GRT and 29 contralateral eyes without disease were included as cases and controls, respectively. Four contralateral eyes were excluded due to a history of RRD-related GRT. The mean time with GRT before surgery was 2.36 ± 1.22 weeks, and patients were followed for an average of 27.20 ± 15.80 months. All other data are listed in Table 1.

**Table 1:** Summary of demographic characteristics and preoperative clinical data of treated and untreated fellow eyes

Characteristics	Control contralateral eyes (n = 29)	Treated eyes (n = 33)	p value
Age (mean ± SD)	55.18 ± 10.31	–	–
Female (N, %)	26 (78.7)	–	–
Preoperative BCVA (logMAR) (median, min–max)	0.09 (0.00–1.60)	1.90 (1.60–2.0)	< 0.0001*
axial length (mm) (median, min–max)	28.12 (23.21–29.42)	28.10 (26.42–31.26)	0.78
Follow-up (months, mean ± SD)	–	27.20 ± 15.82	–
TCA (mm^2^) (mean ± SD)	0.46 ± 0.09	0.36 ± 0.10	0.0004*
LCA (mm^2^) (mean ± SD)	0.32 ± 0.06	0.19 ± 0.06	0.09
CVI (%) (mean ± SD)	71.2 ± 8.50	53.6 ± 8.81	< 0.0001*
Choriocapillaris flow area (mm^2^)	2.08 ± 0.50	1.70 ± 0.51	0.0053*

*BCVA* best-corrected visual acuity, *CI* confidence interval, *GRT* giant retinal tear, *logMAR* logarithm of the minimum angle of resolution, *SD* standard deviation

*Statistically significant differences

### Visual outcomes after surgery

At the last postoperative follow-up visit, mean BCVA improved by 1.28 logMAR in the PPV + SB group, 1.35 logMAR in the PPV group, and 1.31 logMAR across all GRT eyes (Table 2). No significant differences in postoperative BCVA were found between the surgical groups (p = 0.196). BCVA significantly improved after surgery (p < 0.0001) in both surgical groups and the combined surgical group. However, postsurgical BCVA remained poorer than that in the fellow control eyes in the PPV (p = 0.0068), PPV + SB (p = 0.0047), and combined surgical groups (p < 0.0001).

**Table 2:** Summary of preoperative and postoperative BCVA

Parameters	PPV	PPV + SB	Combined
n	14	19	33
Mean presurgical BCVA (logMAR)	1.91	1.89	1.90
Mean postsurgical BCVA (logMAR)	0.55	0.61	0.59
Mean change in BCVA (logMAR)	−1.35	−1.28	−1.31
p value	p < 0.0001	p < 0.0001	p < 0.0001

*BCVA* best-corrected visual acuity, *logMAR* logarithm of the minimum angle of resolution, *PPV* pars plana vitrectomy, *SB* scleral buckling

### Complications after surgery

Six eyes developed a posterior PVR and required additional surgery. No significant differences were found in BCVA change after surgery (p = 1) or final BCVA (p = 0.941) between eyes that did and did not develop PVR, respectively. Of the six eyes, two underwent PPV alone and four received PPV combined with SB; the differences between the surgical groups were not statistically significant (p = 0.618). Three eyes also developed anterior PVR (one in the PPV group and two in the PPV + SB group), with nine eyes requiring additional surgery (three in the PPV group and six in the PPV + SB group). The proportion of eyes requiring additional surgery was not significantly different between the two surgical groups (21.4% in the PPV group and 31.6%; PPV + SB group, p = 0.801). All eyes received gas tamponade, except for one eye that received silicone oil tamponade in the PPV group (the silicone oil was uneventfully removed after 4 months). The difference in tamponade use between the surgical groups was not statistically significant (p = 0.876).

### CVI and CFA measurements

**Fig. 2:**
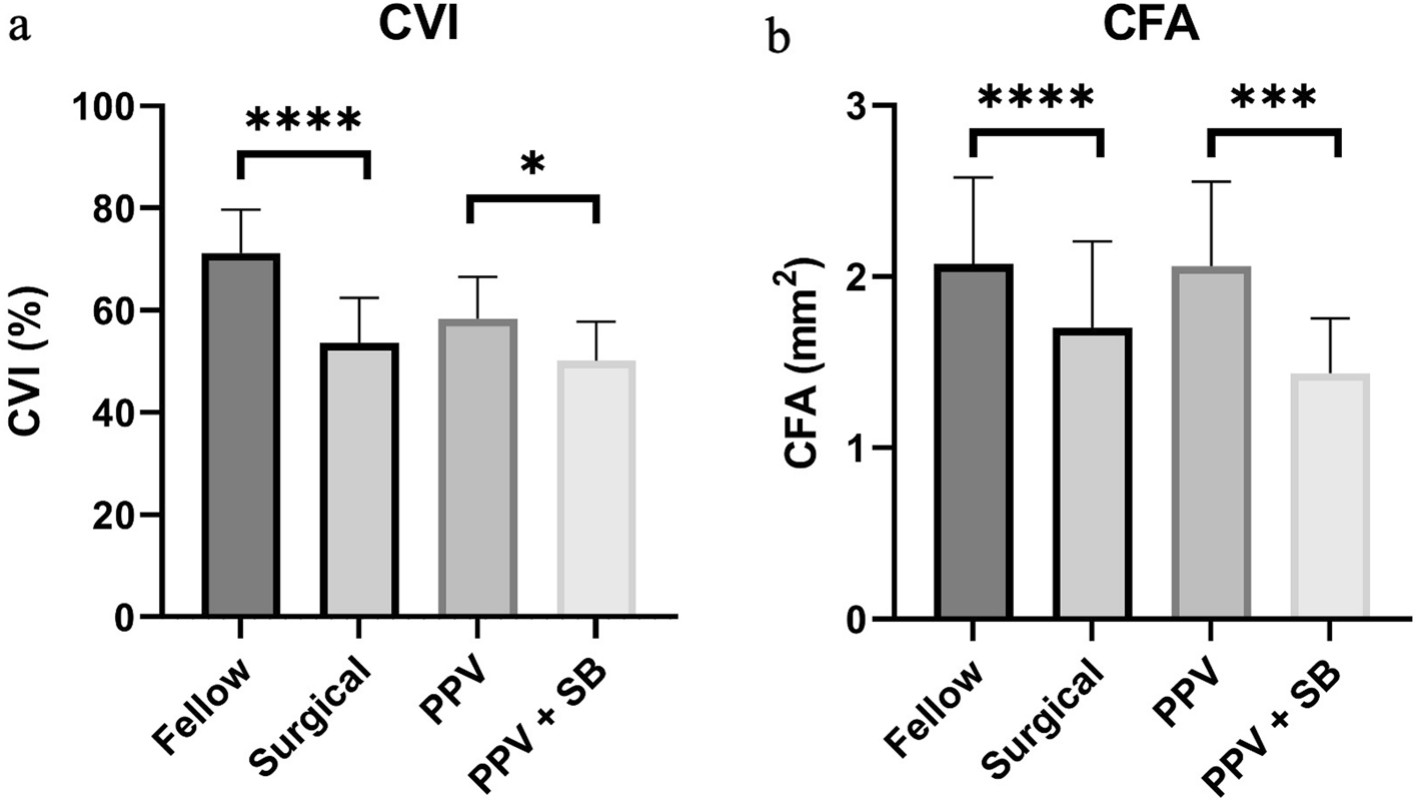
CVI and CFA values across study groups. Optical coherence tomography results were reviewed to obtain **a** CVI values and **b** CFA. Lower CVI and CFA values were found in surgically treated eyes than in their normal fellow eyes. These values were higher in the PPV group than those in the PPV + scleral buckling group. Bars represent the mean ± standard deviation; *p ≤ 0.05, *** p ≤ 0.001, ****p ≤ 0.0001

Compared with contralateral control eyes, surgically treated GRT eyes had significantly smaller LA, TCA, CVI, and CFA values (all p < 0.0001) (Fig. 2). No significant differences were found in LA and TCA values between the PPV and PPV + SB groups (both p > 0.05). However, the PPV + SB group had significantly lower CVI (p = 0.0125) and CFA (p = 0.0003) values than the PPV group (Table 3).

**Table 3:** Mean choroidal measurements across the treated eyes (n = 33), and control eyes values

Parameters	PPV group	PPV + SB group	p value between groups	Overall findings	p values compared with fellow eyes
n	14	19		33	29
LA (mm^2^)	0.212	0.179	p > 0.05	0.193	p < 0.0001
TCA (mm^2^)	0.361	0.365	p > 0.05	0.364	p < 0.0001
CVI (%)	58.4	50.1	p < 0.0125	53.6	p < 0.0001
CFA (mm^2^)	2.06	1.44	p < 0.0003	1.70	p < 0.0001

*LA* luminal area, *TCA* total choroidal area, *CVI* choroidal vascularity index, *CFA* choriocapillaris flow area

### Correlation between choroidal biomarkers and visual function

**Fig. 3:**
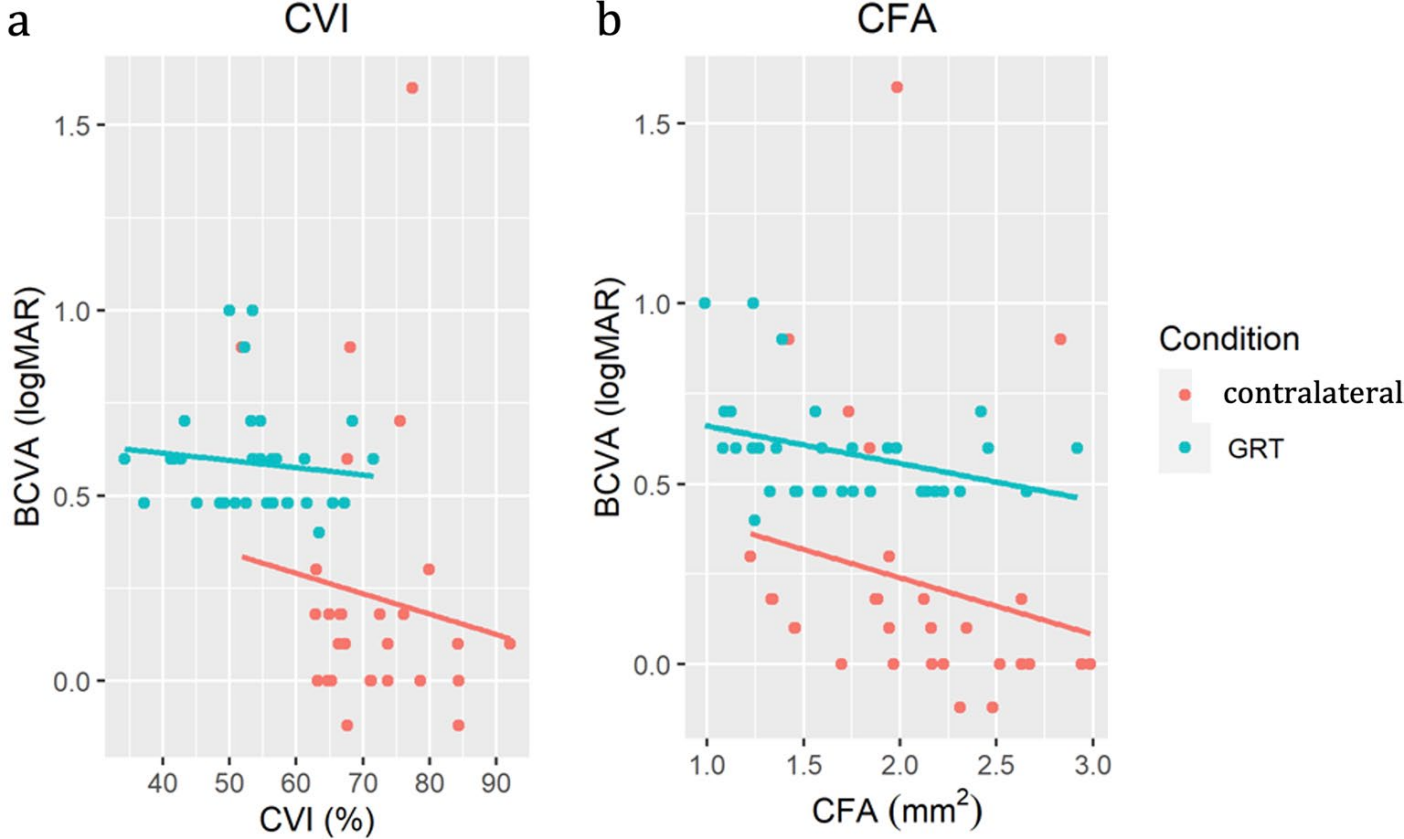
Correlation between CVI, CFA, and BCVA. The postoperative **a** choroidal vascularity index (CVI) or **b** choriocapillaris flow area (CFA) were plotted along the x-axis, while the postoperative best-corrected visual acuity (BCVA) was plotted on the y-axis. The colored lines depict the linear regression of the data for either the fellow eyes or the GRT eyes. All regression lines had negative slopes, indicating that the CVI and CFA were negatively correlated with BCVA

As the state of the choroid may influence visual function, a correlation analysis between choroidal parameters (CVI and CFA) and BCVA was performed. Negative correlations were observed between BCVA, CVI, and CFA (Fig. 3). Since BCVA was measured in logMAR units, this suggests a positive correlation between the two choroidal parameters and visual function; *that is,* a higher CVI or CFA was correlated with better BCVA. CVI and CFA were negatively correlated with BCVA (p = 0.0002 and p = 0.0027, respectively).

A multivariate linear regression model was created to identify biomarkers that influenced visual function. Three anatomical biomarkers were selected, namely CVI, CFA, and axial length. Of the three biomarkers, only the CFA coefficient was significantly nonzero (p = 0.03) and negative (Table 4), which was consistent with the correlation analysis.

**Table 4:** Results from the linear regression model of BCVA

Coefficients	Estimate	p value
Intercept	1.55	0.0297
CVI	0.000226	0.941
CFA	−0.125	0.0321
Axial length	−0.00271	0.237

*CFA* choriocapillaris flow area, *CVI* choroidal vascularity index

**Fig. 4:**
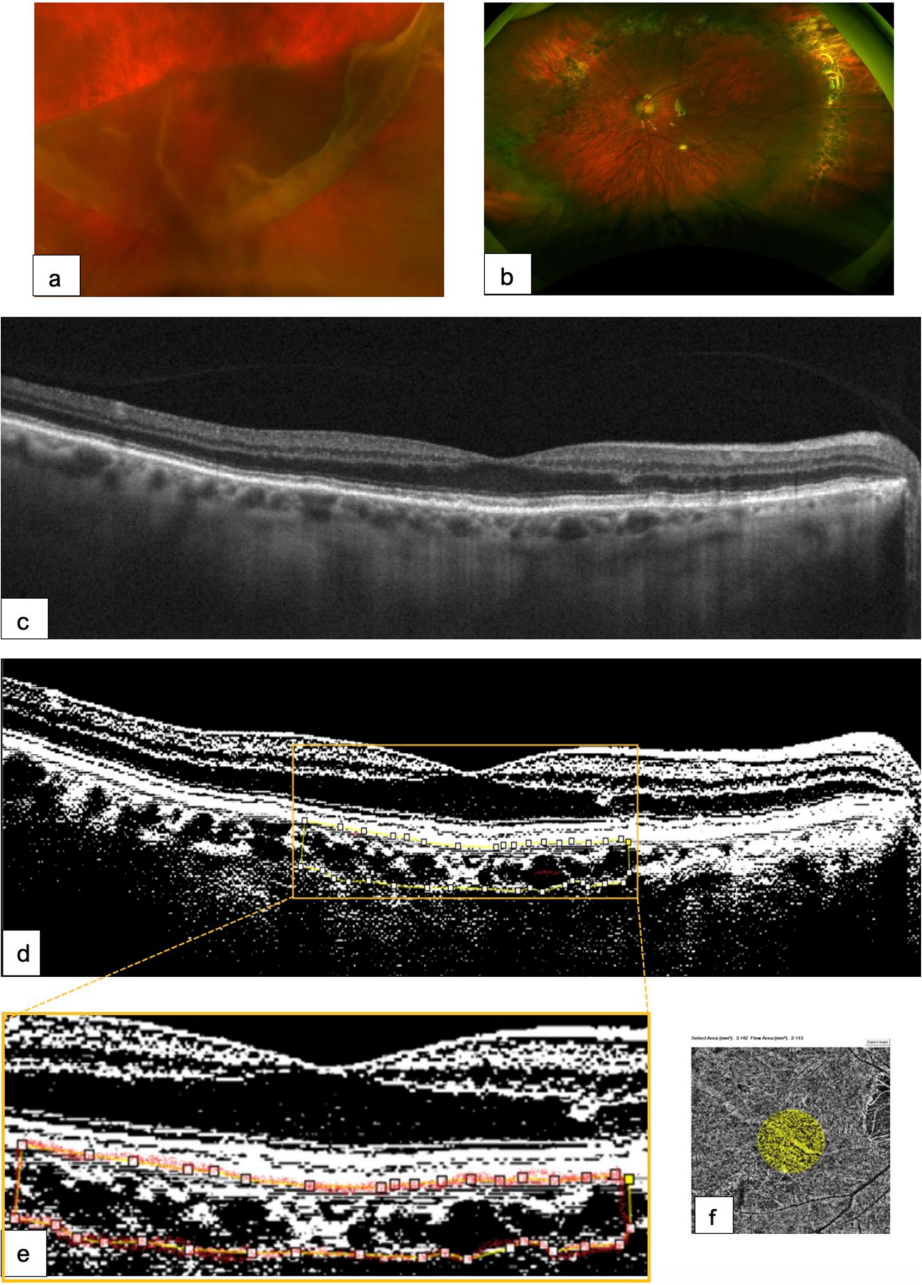
Surgical case 1. Images showing **a** retinal detachment from the giant retinal tear extending from IX to II of the fundus. **b** Nine-month postoperative image after PPV + SB (medium-lying oval sponge) **c** Postoperative OCT with well-defined outer retina layer biomarkers, irregular space at Henle´s layer, no residual subretinal fluid, and well-defined choroidal vessels. **d** The corresponding binarized processing image depicts a normal relationship between the total choroidal area (TCA) and luminal area (LA). The yellow dotted line indicates the selected subfoveal binarized area. The choroidal vascularity index (CVI) was lower than that in the contralateral eye. **e** Magnified image from the yellow inset depicting a preoperative choroidal vascularity index (CVI) of 47.2% calculated within the area clearly delineated by the yellow–red dotted line. **f** The choriocapillaris binarized image depicts a normal postoperative CFA of 2.113 mm^2^ in a selected subfoveal flow area of 3.142 mm^2^. This modified figure was adapted from [31] and used under Creative Commons Attribution 4.0, International License (https://creativecommons.org/licenses/by/4.0/)

**Fig. 5:**
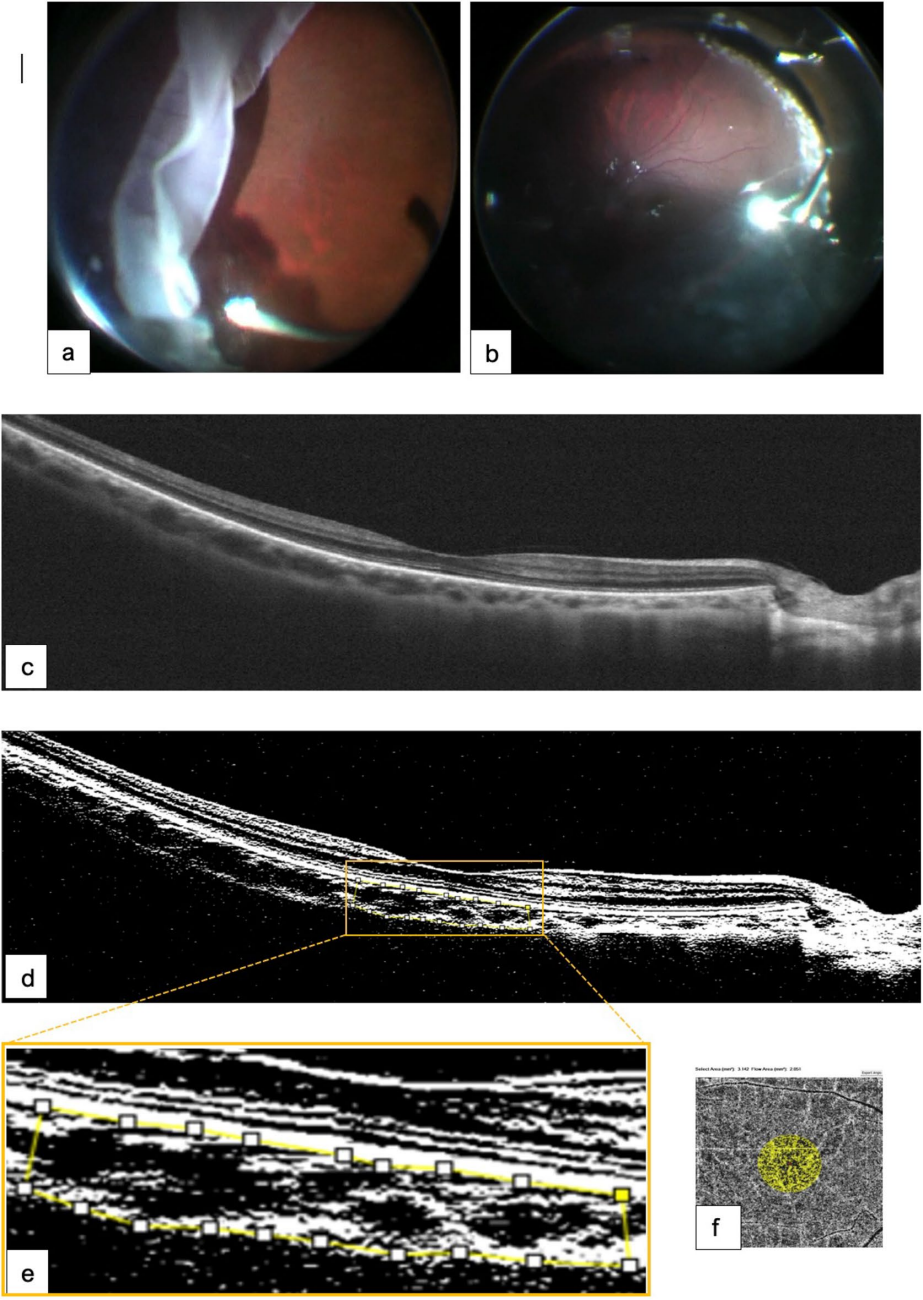
Surgical case 2. Images showing **a** a surgical image of a GRT-associated macula-off RRD that underwent uneventful gas vitrectomy surgery without scleral buckle placement. **b** Image of the retina completely reattached showing peripheral laser spots over the edge of the retina. **c** A 16-month postoperative image showing a visual acuity of 20/40 (logMAR, 0.30). **d**, **e** Binarized images of the choroidal stroma and luminal vascular visualization of the subfoveal choroidal vessels. The yellow dotted line delineates a CVI of 54.9% lower than the one in the fellow eye. **f** Corresponding CFA of 2.051 mm^2^ in the subfoveal flow area of 3.142 mm^2^. This modified figure was adapted from [31] and used under Creative Commons Attribution 4.0, International License (https://creativecommons.org/licenses/by/4.0/)

**Fig. 6:**
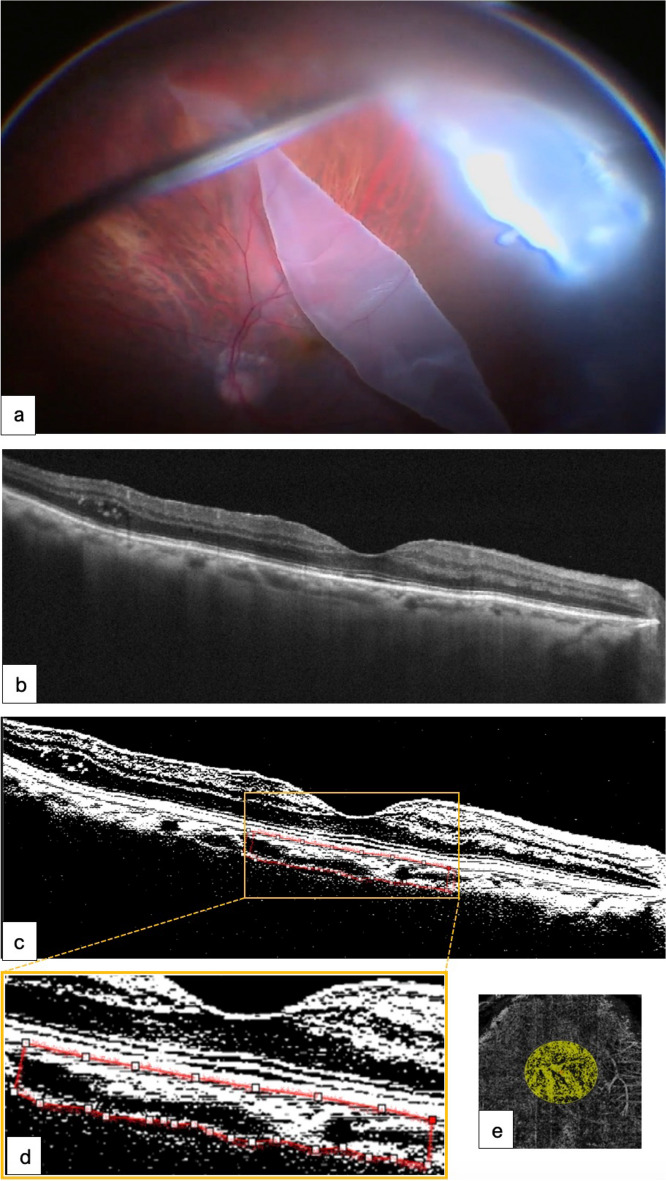
Surgical case 3. Images showing **a** a representative image of a GRT-associated RRD extension of > 180°. The patient underwent uneventful gas vitrectomy. **b** Postoperative image showing an irregular foveal profile with identifiable inner and outer biomarkers and no residual subfoveal fluid. **c** Postoperative 13-month binarized image with perfusion indices lower than those of the fellow eye. **d** The magnified image from the yellow inset depicts the red dotted line that clearly delineates the selected subfoveal area. The CVI was 57.94%, which was lower than that of the normal fellow eye. **e** Image depicting an abnormal postoperative CFA area of 1.682 mm^2^. This modified figure was adapted from [31] and used under Creative Commons Attribution 4.0, International License (https://creativecommons.org/licenses/by/4.0/)

Figures 4, 5 and 6 illustrate representative surgical cases.

## Discussion

Identifying the role of perfusion changes in the choroid and retina in patients with retinal disease is useful for disease diagnosis and management planning. There is a continuing interest in investigating choroidal perfusion in eyes with retinal diseases, given the vital roles of choroids in normal physiology and disease pathology. Previously, detailed imaging and analysis of the choroid were unavailable because of technological limitations; *for example*, dye-based approaches such as indocyanine green angiography can only visualize the frontal choroid [39]. Recent technological advances, specifically the introduction of OCT-A, have provided new opportunities to investigate the choroidal vasculature. OCT-A is particularly transformative because it enables the noninvasive visualization of deep structural and perfusional changes in the retina and choroid.

More recently, Agrawal et al. [30] described CVI as a novel and stable biomarker for the state of choroidal vasculature. Since then, a number of studies have evaluated CVI as a biomarker for vascular function and management of posterior eye conditions [37, 40–43]. An increase in CVI indicates higher perfusion due to a greater vessel diameter or number of vessels in the choroid, whereas a decrease in CVI suggests ischemia from reduced perfusion. Collectively, CVI can be used to assess posterior vascular health, particularly in the context of disease management.

To our knowledge, this is the first study in which CVI data was collected and correlated with postoperative visual outcomes in patients who underwent GRT. Our findings showed that CVI and visual outcomes (measured using BCVA) were positively correlated in the study cohort. Eyes with a higher CVI had better postoperative BCVA scores (lower logMAR values) than did those with a lower CVI. Additionally, non-buckled eyes also had better choroidal flow, as indicated by higher CVI values, and slightly superior visual outcomes (1.35 logMAR improvement with PPV versus 1.28 logMAR improvement with PPV + SB). Contrary to several recently published studies [44–46], we found a statistically significant correlation between the postoperative CVI and BCVA. However, it is worth noting that the correlation is marginal, suggesting that CVI is a relatively weak predictor of visual outcome.

We also compared choroidal biomarkers between eyes with GRT and the control eyes. Notably, postoperative GRT eyes, particularly eyes that underwent vitrectomy combined with SB, had significantly lower LA, TCA, CVI, and CFA values than fellow control eyes. Lower postoperative CVI, with respect to the controls, has previously been reported following posterior eye surgeries [47–49], possibly due to the resolution of inflammation or other triggers that initially increased CVI in the first place. Our findings for buckled eyes contrasted with a recent report in which Bernabei et al. [33] found no difference in CVI postoperatively between control and operated eyes. Specifically, our findings suggest that the mechanical force exerted by the buckle could potentially lead to a higher pressure, which may prohibit the expansion of the LA when compared with non-buckled eyes, thereby lowering the CVI for vitrectomy combined with SB eyes.

CFA is another relevant choroidal biomarker for characterizing perfusional status. Rosenfeld et al. [50] recently examined the relationship between low CFA and visual outcomes, and found a significant correlation between them in eyes with drusen. Our findings in patients with GRT-associated RRD suggest a similar trend in which a significantly positive correlation between CFA and visual outcomes was identified. Another report by Nesper et al. [51] found a significant correlation between the CFA and visual outcomes in patients with reticular pseudodrusen. Collectively, these findings provide insights into the relationship between CFA and visual outcomes and support the hypothesis that CFA is a biomarker of foveal photoreceptor function [50–52].

Utilizing both CFA and CVI may provide a more robust approach for analyzing choroidal blood flow and its impact on visual outcomes. A recent study by Shi et al. [53] revealed that geographic atrophy in eyes with AMD is correlated with both CVI and CFA. Additionally, Wu et al. [54] found that lower CVI and lower flow voids in the choriocapillaris (lower CFA) were both directly related to poor visual outcomes, which is consistent with our findings. We also included CVI, CFA, and axial length in a multivariate regression model for BCVA and found that only CFA had a statistically significant non-zero coefficient. These findings suggest that CFA may be a better biomarker of visual outcomes, at least in eyes with GRT-associated RRD.

Collectively, our findings demonstrate that posterior surgical procedures can affect choroidal blood flow and perfusion status. Specifically, SB placement was found to have a negative effect on choroidal blood flow, as indicated by lower CVI and CFA. Although not statistically significant, a higher percentage of eyes that received SB developed additional complications and required secondary surgery. These results indicate that care should be taken during and after posterior eye surgery to reduce the impact of choroidal blood flow, which plays an important role in maintaining normal photoreceptor function and visual outcomes. Choroidal blood flow biomarkers such as CVI and CFA can provide relevant insights into ocular health and may be used in the management of retinal diseases.

It is worth noting that the present study had some limitations, including its retrospective design and small sample size. In addition, the patients were not randomly selected to undergo SB placement, which added confounding variables to this dataset and reduced the reliability of the results; no statistical adjustments were performed to account for confounding factors. Therefore, these findings should be viewed as hypothesis forming, and additional large prospective studies should be conducted to evaluate our main conclusions.

However, these limitations may be compensated for by the strengths of this study: (1) the relatively long-term follow-up of the study cohort; (2) the limited number of functional and perfusional studies currently available that have investigated choroidal blood flow in patients with GRT-associated RRD surgically treated with vitrectomy (with or without SB); and (3) data from the surgical group were compared with those from the normal contralateral eyes, which was a major strength of the study, as we were able to determine the differences more precisely. Overall, this study provides new insights into the therapeutic management of GRT-associated RRD, and may facilitate future progress in the utility of choroidal biomarkers in patient care.

## Conclusions

In conclusion, choroidal blood flow and perfusion status may be biomarkers of visual outcomes following posterior eye procedures. In this long-term follow-up study of patients with GRT-associated RRD, buckled eyes displayed lower CVI and CFA than non-buckled eyes. CVI and CFA correlated well with BCVA; higher choroidal measurements were associated with better vision. Adjustments for confounding factors beyond subgroup analysis were not feasible in this analysis. Then, those factors should be considered before drawing these conclusions. Finally, our results suggest that buckling should be used cautiously in a limited number of cases with high risk factors because of its potential negative impact on choroidal vasculature, which may adversely affect vision. Further studies should verify the relationship between these biomarkers and visual outcomes.

## Supplementary Information

**Additional file 1.** Giant retinal tear_choroidal vascularity index_Data analysis.R.

## Data Availability

The datasets used in this study have been included in the main text. Photographs and figures from this study may be released via a written application to the Photographic Laboratory and Clinical Archives Department of the Retina Specialists Unit at Oftalmologia Integral ABC, Medical and Surgical Assistance Institution (nonprofit organization), Av. Paseo de las Palmas 735 suite 303, Lomas de Chapultepec, Mexico City 11000, Mexico and the corresponding author upon request. All analysis files and figures (pdf, eps, tiff) can be found in the supplementary file docx.

## Abbreviations

C_3_F_8_: Perfluoropropane
BCVA: Best-corrected visual acuity
CFA: Choriocapillaris flow area
CVI: Choroidal vascularity index
GRT: Giant retinal tear
ILM: Internal limiting membrane
LA: Luminal area
OCT: Optical coherence tomography
PVR: Proliferative vitreoretinopathy
RD: Retinal detachment
RRD: Rhegmatogenous retinal detachment
RPE: Retinal pigment epithelium
SB: Scleral buckling
SD: Standard deviation
SOSR: Single operation success rate
